# Supplementary material for: High molecular weight glutenin gene diversity in Aegilops tauschii demonstrates unique origin of superior wheat quality
Source: Commun Biol. 2021 Nov 1;4:1242. doi: 10.1038/s42003-021-02563-7 (PMC8560932; doi:10.1038/s42003-021-02563-7)
Supplement: Supplementary file 2 — Supplemental Information [file 42003_2021_2563_MOESM2_ESM.pdf]

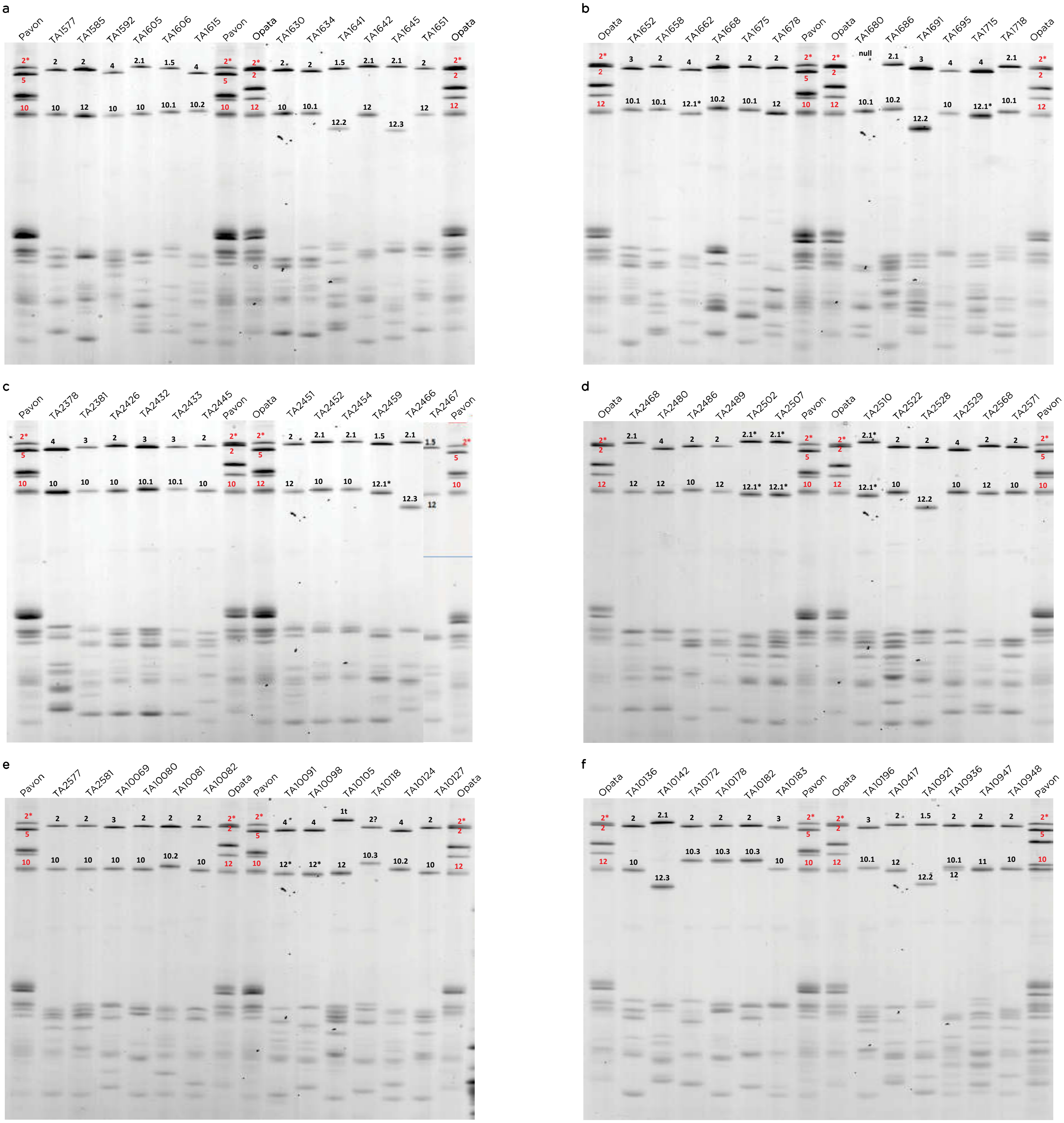

Supplementary Figure 1. SDS-PAGE images. Seventy two *Ae. tauschii* accessions were selected for SDS-PAGE analysis. Hexaploid wheat variety standards Pavon (*Glu-D1 5+10*) and Opata (*Glu-D1 2+12*) were used. Both varieties carry the *Glu-A1 2** x subunit allele that has similar mobility as the x subunit in *2+12.* The final two lanes of **c** were run a separate gel and shown alongside the rest of gel c for brevety.

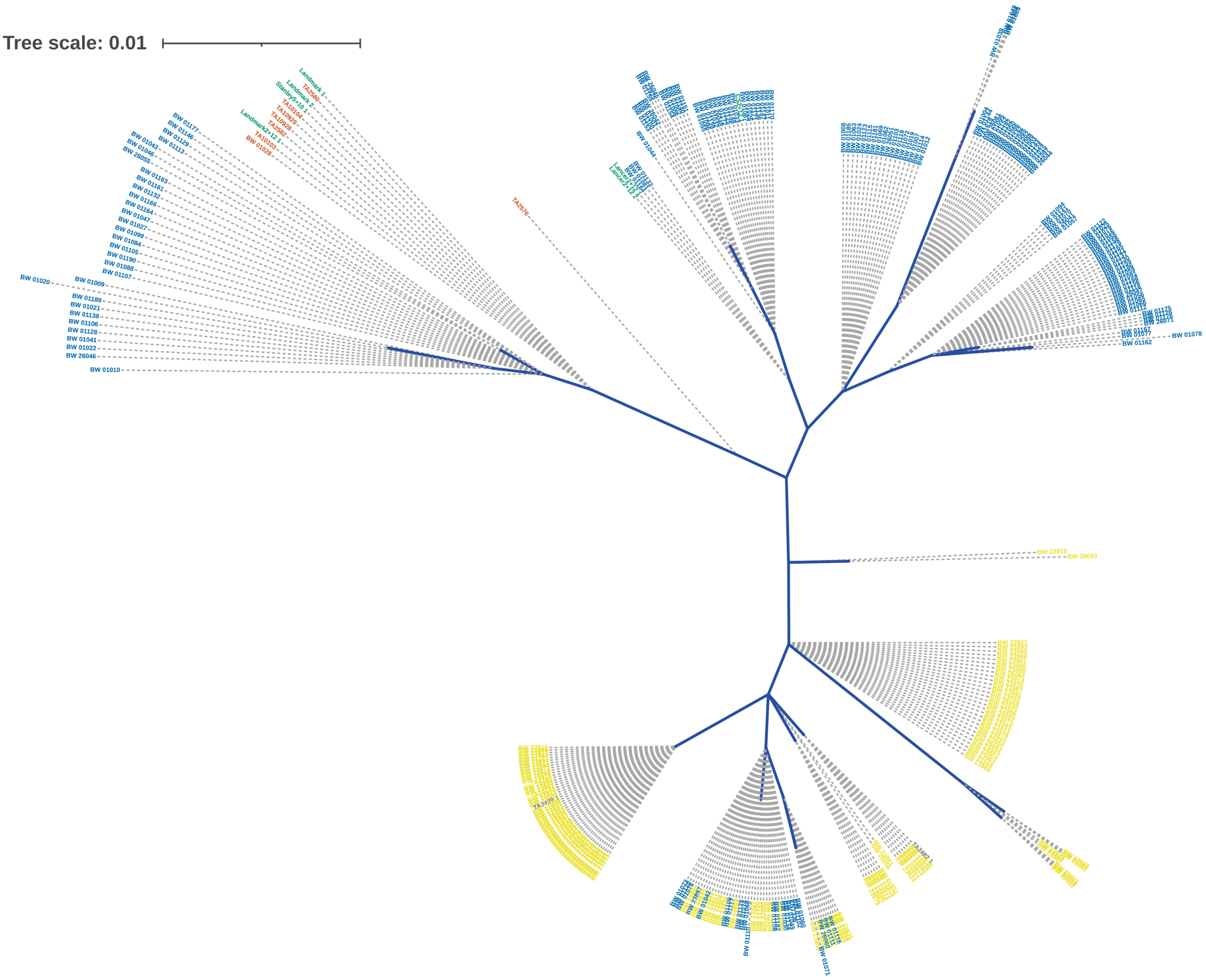

Supplemental Figure 2: *Glu-D1x* **deduced amino acid phylogenic tree.** Amino acid sequences were deduced from single nucleotide variants within the coding region of *Glu-D1x.* The corresponding lineage of each accession is colored in yellow (Lineage 1), blue (Lineage 2), orange (Lineage 3), and green (bread wheat). Interactive tree accessible at https://itol.embl.de/tree/70179110196361341622756779.

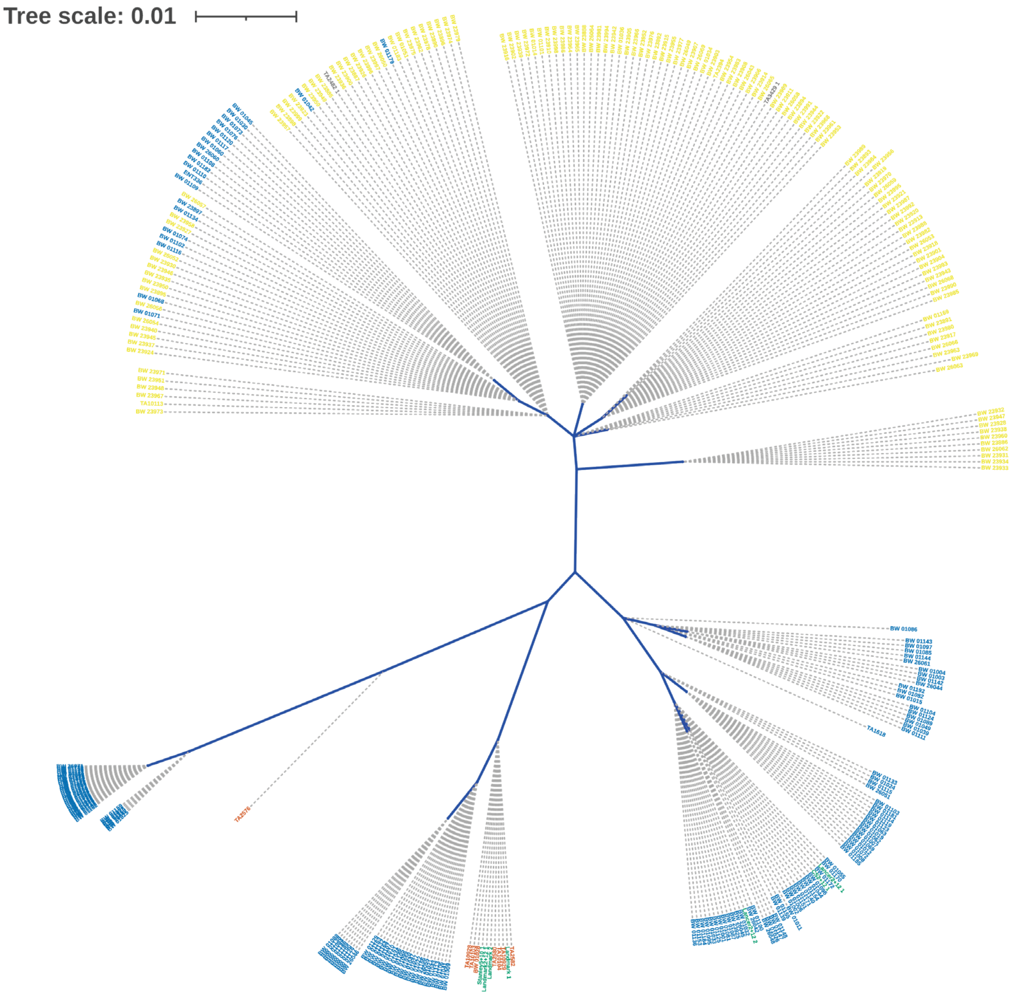

Supplemental Figure 3: *Glu-D1y* deduced amino acid phylogenic tree. Amino acid sequences were deduced from single nucleotide variants within the coding region of *Glu-D1y.* The corresponding lineage of each accession is colored in yellow (Lineage 1), blue (Lineage 2), orange (Lineage 3), and green (bread wheat). Interactive tree accessible at https://itol.embl.de/tree/70179110196462591622758174.
