# Supplementary material for: High molecular weight glutenin gene diversity in Aegilops tauschii demonstrates unique origin of superior wheat quality
Source: Commun Biol. 2021 Nov 1;4:1242. doi: 10.1038/s42003-021-02563-7 (PMC8560932; doi:10.1038/s42003-021-02563-7)
Supplement: Supplementary file 3 — Description of Supplementary Files [file 42003_2021_2563_MOESM3_ESM.pdf]

Description of Additional Supplementary Files

File name: Supplemental Data 1

Description: Details of samples in this study.

File name: Supplemental Data 2

Description: Variant call format file of Ae. tauschii and wheat samples for Glu-D1 in Aet v4genome assembly.

File name: Supplemental Data 3

Description: Annotated variants of Glu-D1 in Aet v4 genome assembly.
